# Pillared and Reduced Graphene Oxide Membranes for Organic Solvent Nanofiltration

**DOI:** 10.1021/acs.iecr.5c01758

**Published:** 2025-09-11

**Authors:** Natechanok Yutthasaksunthorn, Kaung Su Khin Zaw, Scott A. Sinquefield, Sankar Nair

**Affiliations:** † School of Chemical & Biomolecular Engineering, 1372 Georgia Institute of Technology, 311 Ferst Drive NW, Atlanta, Georgia 30332, United States; ‡ Renewable Bioproducts Institute, Georgia Institute of Technology, Atlanta, Georgia 30332, United States

## Abstract

This work demonstrates the capability of pillared and reduced graphene oxide (GO) membranes to perform organic solvent nanofiltration (OSN) in diverse nonpolar and polar solvents. The effects of pillaring by polyconjugated aromatic compounds (PACs) on solvent flux and molecular weight cutoffs (MWCOs) are investigated. Pyranine/solvent green 7 (SG) and toluidine blue O (TBO) were used as pillaring agents, followed by chemical reduction with hydriodic acid. This fabrication process yielded membranes with a stable cross-flow operation in both polar and nonpolar solvents. Pillared and reduced membranes (rSG-GO and rTBO-GO) exhibited 2-fold higher permeances in nonpolar solvents (C_6_–C_10_ alkanes and aromatics) compared to nonpillared membranes, while maintaining MWCOs of 500–600 Da in toluene. The membranes broadly followed a trend of higher permeance with decreasing solvent viscosity but with nuanced deviations based on membrane–solvent interactions. Reducing the thickness to ∼70 nm further enhanced permeance while maintaining rejection of larger solutes.

## Graphical abstract

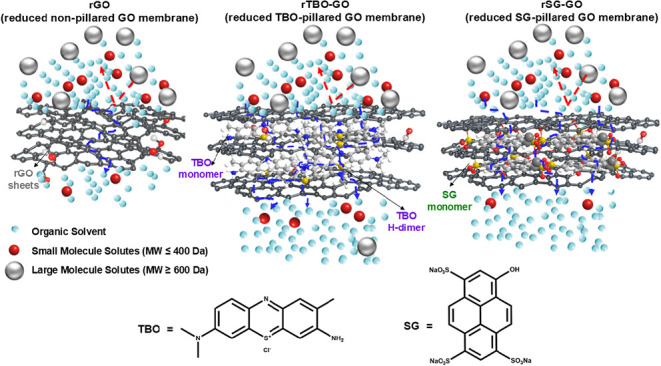

## Introduction

Graphene oxide (GO) and reduced graphene oxide (rGO) nanofiltration membranes have been recognized as a promising alternative to conventional polymeric and ceramic nanofiltration membranes for water treatment and other aqueous-phase separations,^1–8^ due to their tunable performance and excellent chemical stability under harsh conditions of extreme pH, temperature, and solute concentrations. GO-based membranes have three distinct forms: assembled GO laminates, nanoporous (perforated) graphene/GO layers, and composites of GO with other materials. The laminate GO membranes provide selective 2D nanochannels in the interlayer for fast permeation of water spaces while maintaining good mechanical stability.^9–11^ A range of techniques, such as spray-coating, spin-coating, drop-casting, dip-coating, filtration, and layer-by-layer assembly, have been used to prepare GO laminate membranes.^12–15^ Recent efforts have also demonstrated continuous slot-die casting (“roll-to-roll”) methods for production of large-area rGO membranes.^16^ The interlayer spaces and the functional groups on GO nanosheets can be modified (or cross-linked) in multiple ways to control molecular/ionic sieving properties.^11,14,17–19^

However, there are many applications in which nanofiltration must be carried out in nonaqueous solvents, i.e., organic solvent nanofiltration (OSN). This is a rapidly growing research area in membrane science with many studies in recent years on the development of polymeric and composite membranes.^20^ Due to the general susceptibility of polymeric membranes to damage by different types of organic solvents, covalent cross-linkers have been used to improve their chemical resistance and antiplasticization properties.^21–23^ While polymeric OSN membranes are available for use in polar solvents (such as alcohols, acetone, and methanol), they have greater limitations in terms of solvent compatibility and stability, especially in hydrocarbon streams.^24–26^ There are a number of challenging applications in this area that currently cannot be accessed, such as fractionation/deasphalting of crude oils and bio-oils, and nanofiltration of refinery products. GO-based membranes could be promising candidates for hydrocarbon OSN due to their excellent mechanical strength, thermal and chemical stability, and tunable pore size. However, a major challenge for GO membrane OSN for hydrocarbon solvents is to increase the hydrophobicity/organophilicity of the membrane while maintaining its porosity and structural stability.^27,28^ Simple chemical or thermal reduction of GO to rGO increases the hydrophobicity but also leads to the collapse of the GO nanosheets toward the graphite structure, hence greatly reducing (or eliminating) the membrane porosity and permeability.^29–31^

A potential solution is to first “pillar” the interlayer spaces of GO membranes using specific intercalants and then perform controlled reduction to obtain organophilic membranes that retain porosity in the interlayer space due to the pillaring.^3,32–35^ A particularly attractive approach is to intercalate the interlayer spaces with polyconjugated/polycyclic aromatic compounds (“PACs”), which are a large class of molecules and organic ions, including several types of dyes and pigments. These molecules/ions can bind very strongly to graphenic/GO surfaces primarily due to π–π interactions and secondarily due to electrostatic interactions.^2,5,36,37^ A few reports have studied in detail the intercalation of PACs of different molecular classes into GO and rGO membranes, such as (7-amino-8-methylphenothiazin-3-ylidene)-dimethylammonium chloride (known as toluidine blue O, or TBO)^2,38^ and trisodium 8-hydroxypyrene-1,3,6-trisulfonate (known as pyranine or solvent green 7, SG7)^5^ (Figure 1). These PACs led to specific modifications in the water flux and solute rejections. In the case of TBO, multiple membrane microstructures were obtained containing varying amounts of monomeric, dimeric, and oligomeric TBO aggregates pillaring the interlayer spaces, which in turn led to nonmonotonic trends in fluxes and solute rejections as a function of TBO loading.^2,38^ In the case of SG7 intercalation,^5^ monomeric pillaring was assumed since there was no evidence for the formation of SG7 aggregates.

**Figure 1:**
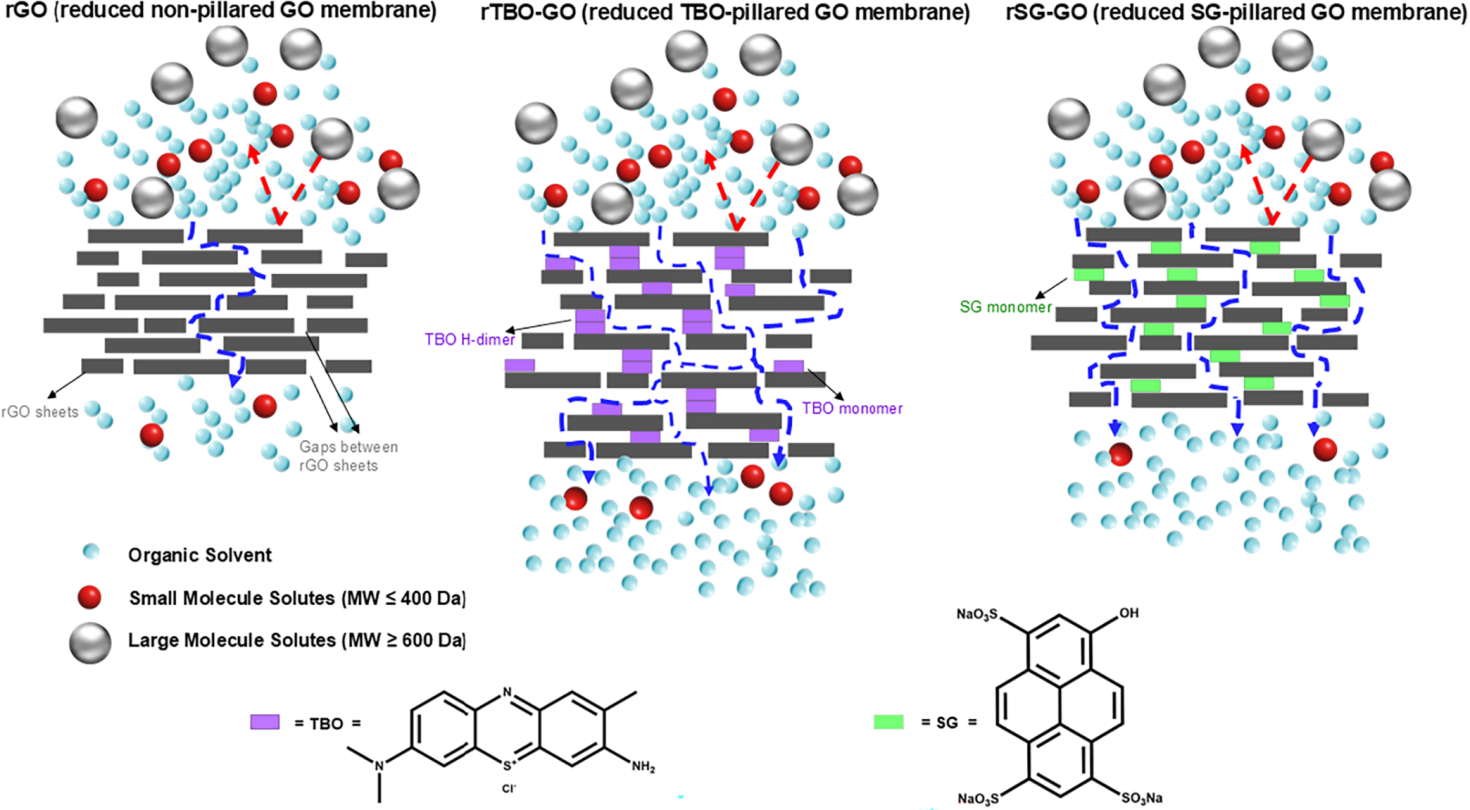
Schematic showing the pillaring and reduction approach to the fabrication of organophilic GO-based membranes with two polyconjugated aromatic compounds (SG and TBO) as intercalant species used in this work.

The objective of this paper is to investigate the use of the above two PAC intercalants combined with chemical reduction of the resulting intercalated GO membranes and to determine whether such “pillared-reduced” membranes can allow significant fluxes of hydrocarbon solvents and organic solvent nanofiltration (OSN) capabilities. We fabricate rGO membranes (Figure S1, Supporting Information) intercalated with TBO or SG7, both of which have large planar aromatic, polyconjugated structures that strongly anchor them on the graphenic regions of GO sheets via π–π interactions. We use poly­(vinylidene fluoride) (PVDF) porous polymer substrates with a nominal pore size of 0.03 μm for membrane fabrication, due to the excellent hydrocarbon resistance of PVDF. Among several available methods for GO reduction,^39–45^ we selected chemical reduction with hydriodic acid (HI) (Figure S2), which can effectively reduce all three main oxygenated functional groups (carboxyl, hydroxyl, and epoxy) via a proton-transfer mechanism (Figure S3). HI offers strong reductive ability under mild room-temperature conditions, minimizing structural damage while enabling a cost-effective, scalable process. Its controllability allows tuning of the reduction degree through adjustment of the concentration or reaction time. The OSN performance of the membranes was evaluated using a range of hydrocarbon-soluble molecules in toluene, selected to span a broad range of molecular weights. The solutes included azobenzene (182.8 g mol^–1^), Oil Blue N (378.5 g mol^–1^), Oil Red O (408.5 g mol^–1^), fullerene C_60_ (720.6 g mol^–1^), fullerene C_70_ (840.8 g mol^–1^), and polystyrene standards (2000 g mol^–1^). These molecules enabled a systematic assessment of the membranes’ molecular weight cutoff (MWCO) and rejection behavior in an organic solvent environment.

## Experimental Methods

The Supporting Information gives a complete account of the preparation of the membranes, characterization methods, permeation measurements, and modeling of pore size distributions. Relevant methods from the literature^46–48^ are also cited therein.

## Results and Discussion

Four types of membranes are discussed here: (1) GO membranes—labeled as *GO*, (2) reduced GO membranes via reduction with aqueous HI—labeled as *rGO*, (3) pillared GO membranes—labeled *TBO-GO* and *SG-GO*, and (4) their HI-reduced versions—labeled *rTBO-GO* and *rSG-GO*. Photographs of these membrane coupons (47 mm diameter) are shown in Figure 2a,f. Visual changes after HI reduction reflect modifications resulting from the chemical reduction. The varying coloration highlights distinct interactions between the GO structure and the pillaring agents, which significantly influence the membrane hydrophobicity and interlayer spacing, as verified by subsequent XPS and XRD analyses. All of these membranes were fabricated by vacuum filtration with the same quantity of GO nanoflakes, and all have a nominal thickness of 100–160 nm in the dry state. Variations in thickness between the membranes are due to modification of the interlayer spacing due to pillaring.

**Figure 2:**
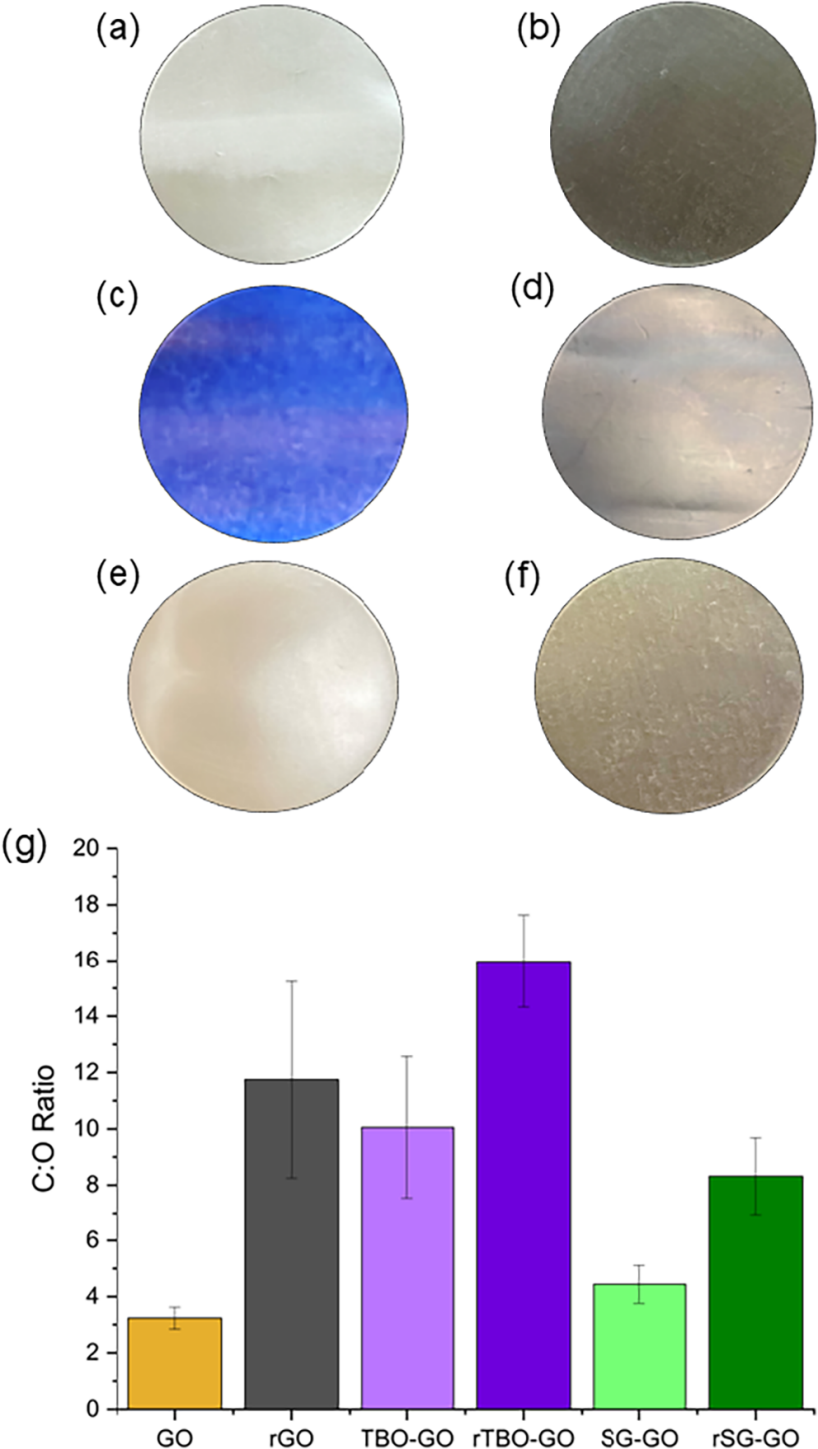
Photographs of GO-based membranes: (a) GO, (b) TBO-GO, (c) SG-GO, and their HI-reduced forms, (d) rGO, (e) rTBO-GO, and (f) rSG-GO (coupon diameter: 47 mm); (g) XPS-determined C/O ratios before and after reduction.

The XPS-derived C/O ratios (Figure 2g) provide quantitative insight into the chemical evolution of GO-based membranes following pillaring and reduction. Example spectra and peak deconvolution are shown in Figure S4. Pristine GO exhibits a low C/O ratio (∼3), characteristic of its highly oxygenated structure. Upon intercalation with conjugated polyaromatic intercalants, the C:O ratio increases, as expected. TBO-GO displays a large increase in the C:O ratio (∼10), whereas SG-GO shows a modest increase (∼4.5). These differences reflect the chemical nature of the intercalants. TBO contains no oxygen, whereas SG has significant oxygen content due to the sulfonate groups. Following HI reduction, the C/O ratios of the nonpillared (rGO) and pillared (rTBO-GO and rSG-GO) membranes increase markedly (to 12, 16, and 8, respectively) relative to their as-made counterparts, indicative of extensive deoxygenation. X-ray diffraction (XRD) analyses were conducted to elucidate interlayer spacing variations among GO-based membranes under different solvent environments, as depicted in Figure 3. These XRD data serve as structural proxies for the corresponding reduced membranes, since post-reduction rGO membranes are known to exhibit a more disordered structure resulting in very broad or undetectable XRD peaks. In their dry state, the pristine GO membrane exhibited a baseline interlayer spacing of approximately 7.5 Å (average), consistent with literature values.^3,5,46^ Upon exposure to polar solvents (water and ethanol), significant swelling of the nonpillared GO membrane was observed, expanding the average interlayer spacing to approximately 13 Å, due to solvent intercalation driven by interactions with hydrophilic oxygen groups. Conversely, immersion in nonpolar solvents such as hexane and toluene resulted in negligible changes in *d*-spacing, suggesting minimal solvent intercalation due to weaker interaction. The introduction of pillaring agents (TBO and SG) significantly modified the membrane structure. The larger dry-state interlayer spacing observed in the TBO-GO membrane (12 Å) compared with the SG-GO membrane (8 Å) is directly attributed to the molecular configuration of the pillaring agents in the interlayer spaces. As we have discovered in recent works, TBO tends to form stacked dimers (H-dimers) at high loadings within GO/rGO membranes, thereby dramatically increasing interlayer spacing.^2,38^ In contrast, SG molecules, containing multiple hydrophilic sulfonate groups (−SO_3_^–^), exhibit negligible stacking and instead adopt mainly a monomeric configuration.

**Figure 3:**
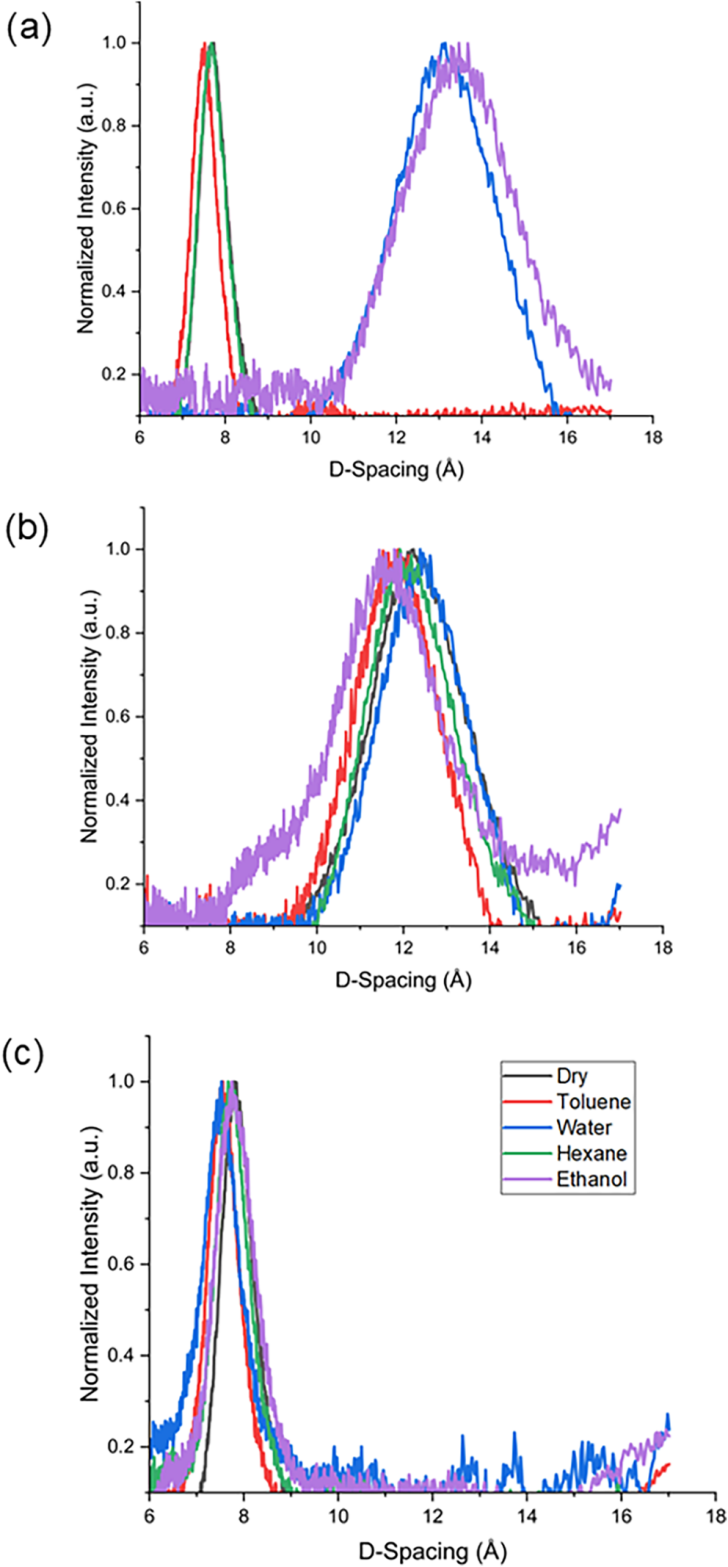
X-ray diffraction (XRD) patterns of interlayer spacing in (a) GO, (b) TBO-GO, and (c) SG-GO membranes under different solvent immersion conditions.

This structural interpretation is corroborated by aqueous-phase UV–vis absorption spectroscopy. The aqueous-phase absorption spectra of TBO (Figure S5a) have been studied in detail experimentally and computationally.^2,38,49^ At low concentrations, only a broad monomeric peak is present. At higher concentrations, a second blue-shifted peak appears, which is due to vertical π–π stacking of TBO (H-dimers). On the other hand, the absorption spectra of aqueous-phase SG (Figure S5b) exhibit several electronic transitions of the monomer. However, no new peaks arise at higher concentrations, clearly indicating that the SG maintains a monomeric form. We speculate that π–π stacking in SG is more hindered than in TBO, due to the much stronger electrostatic repulsion (as well as out-of-plane steric hindrance) of the four sulfonate groups on each pyrene backbone.^50,51^ The UV–vis spectrum of the TBO-GO suspension prior to membrane fabrication (Figure S5c) displayed H-dimer formation, consistent with our previous work.^38^ The UV–vis spectrum of the SG-GO suspension (Figure S5d) is the same as that of monomeric aqueous SG. These findings directly support the proposed interlayer arrangements and the dry-state XRD observations. Notably, both pillared membranes maintained stable *d*-spacing (no significant swelling) across all solvents tested, regardless of the polarity. This highlights the effectiveness of PAC pillaring agents in preventing solvent-induced swelling.

To quantitatively assess the solvent transport behavior, we report both flux (*J*, L·m^–2^·h^–1^) and permeance (*J*/Δ*P*, L·m^–2^·h^–1^·bar^–1^) in the subsequent discussion. The flux directly measures the throughput at a particular transmembrane pressure (TMP), whereas the permeance normalizes the flux with respect to the TMP. The two quantities thus allow more meaningful comparisons across different membranes, solvents, and measurement methods. Figure 4 shows the water flux and dye rejections in all of the membranes. The pristine GO membrane exhibited the highest water flux (Figure 4a). Upon reduction to a rGO membrane, there was a 2-fold reduction in water flux, as expected due to the presence of a greater proportion of reduced/graphenic regions. In contrast, the pillared membranes maintained higher water flux after reduction (rTBO-GO and rSG-GO) relative to the corresponding nonpillared membranes after reduction (rGO). Clearly, pillaring/intercalation facilitates the superior retention of porosity and membrane microstructure postreduction. Figure 4b shows the rejections of dye molecules in the MW range 400–800 Da (see the Supporting Information for further details). Before the reduction process, both the pillared membranes (TBO-GO and SG-GO) showed an estimated molecular weight cutoff (MWCO) of ∼450 Da, slightly smaller than that of the pristine GO membrane (∼470 Da). This is due to the presence of the pillaring agents that retard the transport of bulky dye molecules in the interlayer spaces, through a combination of steric and potentially electrostatic interactions. It is also consistent with their lower water fluxes relative to that of the GO membrane in Figure 4a. Upon reduction, the rTBO-GO membrane exhibited a significantly higher molecular weight cutoff (∼650 Da) compared to its TBO-GO counterpart before reduction. Assuming no significant change in the pillared microstructure between the two membranes, this effect could be attributed to the removal of polar oxygen-containing groups during HI reduction, which weakens interlayer hydrogen bonding and diminishes polar interactions. On the other hand, the reduced rSG-GO membrane did not experience a significant change in MWCO relative to the SG-GO membrane. This is attributed to the chemical stability of the sulfonate groups in SG, which preserve interlayer interactions after reduction. In aqueous systems, dye rejection arises from a complex combination of factors including (i) size exclusion, (ii) electrostatic repulsion, especially from retained sulfonate groups in SG-GO, (iii) π–π interactions between aromatic dyes, GO nanosheets, and pillaring agents, and (iv) steric hindrance modulated by interlayer spacing and degree of reduction. The above findings, while not yet fully understood in detail, emphasize the significant influences of PAC composition as well as microstructural arrangement between the interlayer spaces.

**Figure 4:**
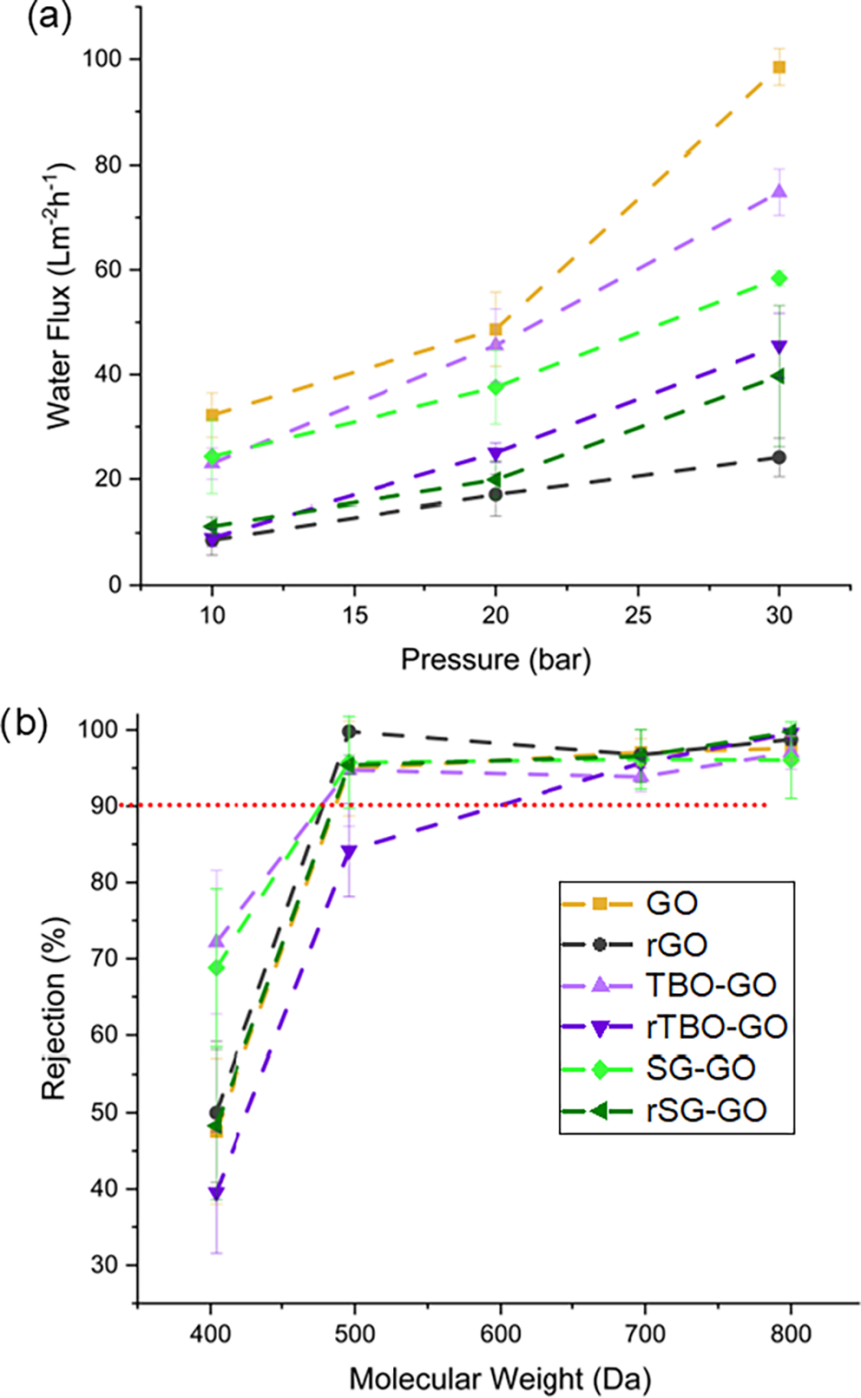
Effect of pillaring/intercalation and reduction on (a) water flux at 25 °C and transmembrane pressure differentials of 10–30 bar and (b) rejections of aqueous dyes at 30 bar.

The organic solvent permeation characteristics and stability of the three reduced membranes (rGO, rTBO-GO, and rSG-GO) were systematically evaluated by continuous cross-flow measurements using a spectrum of organic solvents (nonpolar and polar, aliphatic and aromatic, hydrocarbon and oxygenated) at 10, 20, and 30 bar and 25 °C over a period of 5 days for each type of membrane. Prior to permeation measurements, each membrane sample was initially verified for solvent stability by immersion in ethanol and water for 30 days each (example photographs in Figure S6). For the OSN measurements, the selected solvents are water, ethanol, linear alkanes (*n*C_6_-*n*C_10_), and aromatics (C_6_–C_8_: benzene, toluene, *p*-xylene). Each membrane was mounted in the cross-flow permeation system (Figure S7) and sequentially evaluated with each of the feed solvents. Typically, a solvent required 3–6 h to reach a steady-state permeation flux. The steady-state fluxes for each solvent were recorded at least three times during a sustained steady-state operation period of about 6 h, before moving to the next solvent. This sequential evaluation was then carried out a second time (i.e., two cycles in total). The steady-state data obtained were used to determine the averaged flux values, permeances, and their statistical error bars. The same procedure was used for all three types of membranes. As shown in Figure 5a, all three reduced membranes demonstrated stable and reproducible operation in both alkane and aromatic hydrocarbon solvents, as well as in ethanol and water. In general, the rSG-GO membrane showed significantly higher permeances in hydrocarbon solvents. The rTBO-GO membrane showed lower permeance in solvents with viscosities below 0.5 cP, but comparable or slightly lower permeance than rSG-GO in solvents with viscosities above 0.5 cP. The nonpillared rGO membrane consistently exhibited the lowest permeances across both polar and nonpolar solvents. To further verify the chemical stability and retention of the pillaring agents under these operational conditions, UV–Vis spectroscopy was performed on the cumulative permeates collected after the extended nanofiltration measurements in water and toluene. No characteristic absorbance peaks corresponding to TBO or SG were detected, indicating the negligible leaching of PACs and confirming their stability within the membrane (Figure S8).

**Figure 5:**
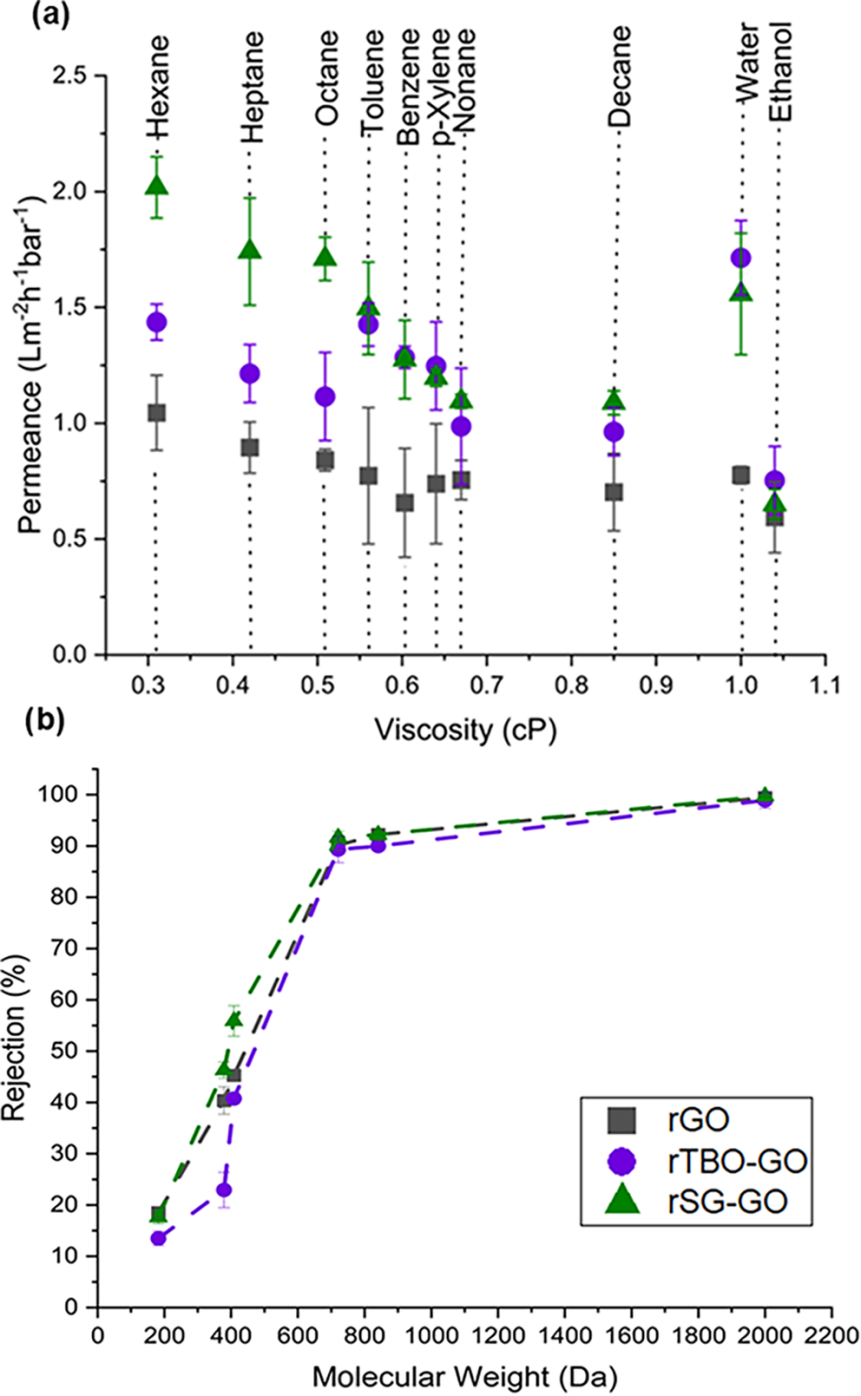
Organic solvent permeation and dye rejection characteristics of reduced pillared and nonpillared membranes. (a) Pure solvent permeance as a function of solvent viscosity. (b) Rejection of hydrocarbon-soluble dyes and bulky hydrocarbon solutes at 30 bar in toluene solvent as a function of molecular weight.

Overall, the permeance trends of the organic solvents (*i.e*., excluding water) in all three membranes are influenced by pressure-driven “viscous flow” through the interlayer spaces, with a clear inverse relationship between permeance (flux/TMP, in liter·m^–2^·h^–1^. bar^–1^) and solvent viscosity. However, there are further nuances in the permeance behavior that are expected to be caused by specific interactions between the solvent molecules, the rGO surfaces, and the pillaring agents. For example, the region of 0.5–0.7 cP (containing both alkanes and aromatics) shows nonmonotonic behavior in the case of rTBO-GO and rGO membranes, but broadly monotonic behavior in the case of the rSG-GO membrane. At present, detailed explanations of these nuanced trends in the observed behavior are not available. However, these observations clearly indicate the role of solvent–membrane interactions within the pillared microstructure. These interactions are expected to depend strongly on the shape, size, and chemical functionality of the solvent molecules. Additionally, the slope of the permeance-viscosity dependence differs markedly between polar and nonpolar solvents, underscoring the influence of solvent–membrane interactions beyond simple viscous flow. While the present paper is mainly concerned with nonpolar hydrocarbon solvents, the overall trends are also consistent with prior measurements in polar organic solvents for polymeric membranes and GO-based membranes.^26,46,52,53^

MWCO measurements were conducted by using dyes and large hydrocarbon solutes of varying molecular weights at a concentration of 20 ppm in toluene. As shown in Figure 5b, the reduced pillared membranes are effective in rejecting these solutes. The rTBO-GO membranes consistently exhibited higher MWCO values than the rGO and rSG-GO membranes in both toluene (Figure 5b) and water (Figure 4b), clearly indicating a more open interlayer structure. Slightly higher MWCOs were observed in toluene compared to water, likely due to reduced interlayer interactions in nonpolar environments. In addition, the absence of electrostatic interactions with neutral solutes (e.g., fullerene C_60_, Oil Red O, and polystyrene) reduces surface-chemistry-driven selectivity, resulting in molecular sieving being the more dominant rejection mechanism. The more sharply defined MWCOs and improved fluxes in rTBO-GO and rSG-GO support the role of PAC pillaring in preserving interlayer architecture upon reduction, thereby enabling both higher permeance and selectivity. Despite its higher solvent permeance, rSG-GO displayed a lower MWCO than rTBO-GO, suggesting that the lateral arrangements of the SG molecules (versus the vertically stacked arrangements of TBO) create a more effective retardation of bulky solutes. These results align with prereduction XRD data, wherein SG-GO showed a stable lower interlayer compared to TBO-GO and nonpillared GO membranes. Together, the MWCO and permeance data highlight the interplay between membrane chemistry, solvent polarity, and solute–membrane interactions in governing separation performance.

Next, we performed continuous cross-flow nanofiltration measurements in toluene. Initially, we performed nanofiltration of 20 ppm fullerene (C_60_, MW 720 Da) dissolved in toluene, over 120 h of operation at 25 °C and 30 bar TMP (Figure 6a). Both flux and C_60_ rejection (>90%) remained stable throughout the measurements, demonstrating excellent operational stability of all three membranes. Next, we performed nanofiltration of a much more concentrated (1 g/L) solute mixture of 0.5 g/L C_60_ (MW 720 Da) and 0.5 g/L Oil Red O (MW 408 Da) dissolved in toluene, over a period of 40 h. As shown in Figure 6b, the rSG-GO and rTBO-GO membranes maintained high C_60_ rejection (∼90%) and moderate Oil Red O rejection (40–60%) during continuous nanofiltration. The stable rejection trends throughout the 40 h measurement further confirm the robustness of the membranes and the lack of fouling by the solutes or degradation by the solvent.

**Figure 6:**
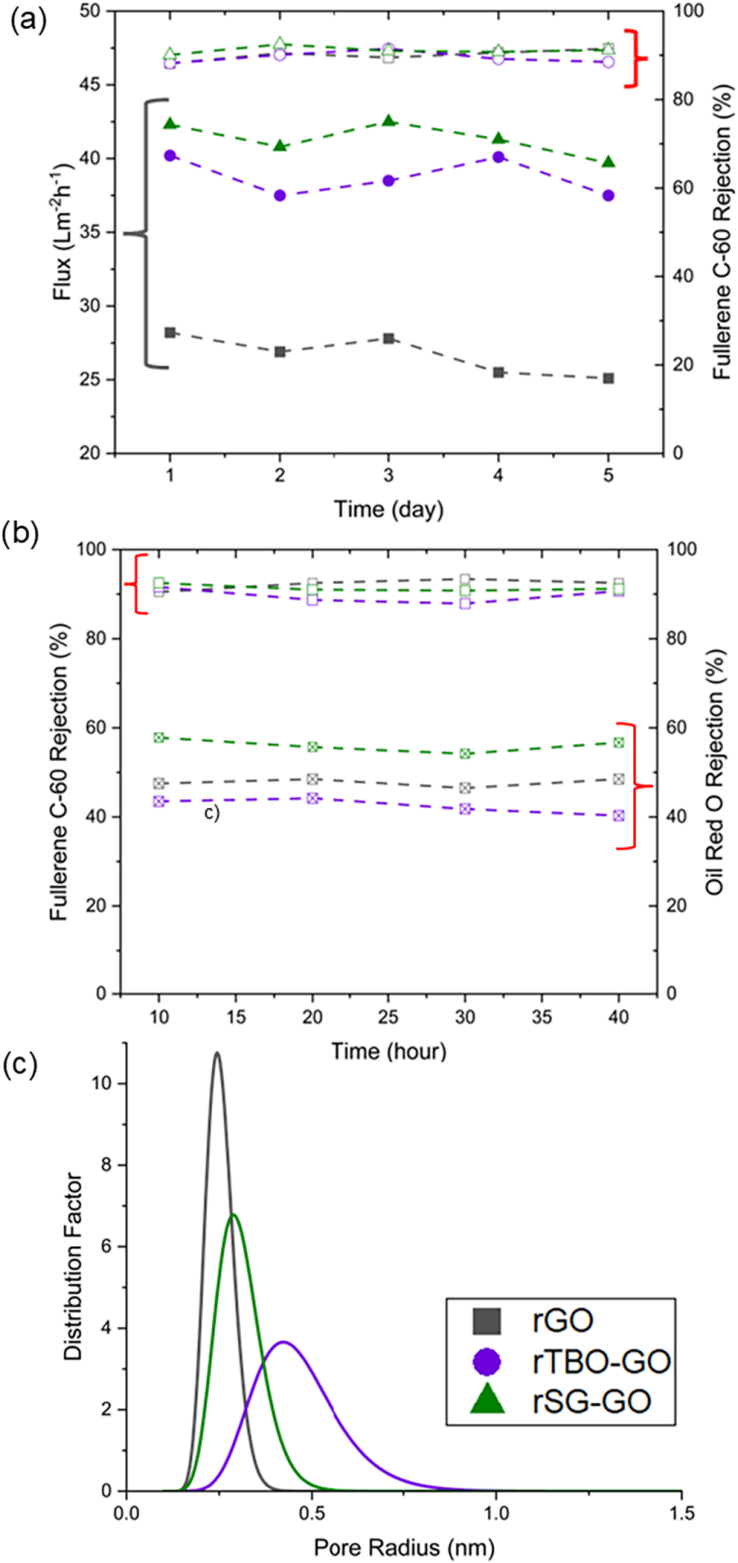
Nanofiltration performance of reduced pillared and nonpillared membranes. Open symbols denote solute rejection. (a) Stability of rGO, rTBO-GO, and rSG-GO membranes during fullerene C_60_ nanofiltration over 120 h. (b) Rejection of a mixture of C_60_ and Oil Red O in toluene over 40 h. (c) Comparative estimates of pore size distributions of the membranes.

Effective pore size distributions (Figure 6c) were derived from the solute rejection and permeation data (see the Supporting Information for the relevant equations and assumptions) and reveal distinct differences among the membranes. It is important to note that the absolute values of pore size obtained by this approach are subject to several simplifying assumptions regarding the properties of the solute, solvent, and the pores. However, the relative comparison between the membranes is informative. The rGO membrane exhibited the tightest pore size distribution and the smallest average pore radius (∼0.25 nm), consistent with its dense, nonpillared structure and relatively lower solvent permeance. The rSG-GO membrane showed a broader distribution centered around ∼0.3 nm, whereas rTBO-GO displayed the broadest distribution as well as the largest average pore radius of ∼0.45 nm. These trends align well with the other characterization data, wherein rTBO-GO exhibits a more expanded and diverse microstructure due to the presence of monomeric and dimeric intercalants, leading to a broader pore size distribution. This also correlates with the higher MWCO and solvent fluxes of rGO-TBO and offering higher flux. The foregoing results illustrate the capability of PAC intercalants for more precise tuning of the interlayer chemistry for organic solvent nanofiltration.

The hydrocarbon permeation and OSN characteristics of the present membranes can be compared with the few recent state-of-the-art membranes addressing hydrocarbon applications. For example, Yoshiwaka et al. reported a toluene permeance of 0.11 L·m^–2^·h^–1^·bar^–1^ with a polyketone composite membrane,^54^ Trivedi et al.^55^ achieved 0.6 L·m^–2^·h^–1^·bar^–1^, and Ma et al. reported up to 20 L·m^–2^·h^–1^·bar^–1^ in a fluorinated polymeric membrane.^56^ Guo et al.^57^ developed a fluorinated membrane with a hexane permeance of 0.3 L·m^–2^·h^–1^·bar^–1^. Most commercial polymeric OSN membranes, despite their gradual adoption, also have limitations, such as swelling, plasticization, and restricted solvent compatibility, particularly in highly nonpolar or aromatic solvents. The rGO-based membranes can offer excellent chemical resilience to hydrocarbons, but are still not well explored for the OSN in hydrocarbon solvents. Recent GO membrane studies primarily reported performance in polar solvents.^58–60^ Yang et al. demonstrated up to 10 L·m^–2^·h^–1^·bar^–1^ hexane permeance in ultrathin (∼10 nm) GO membranes. In comparison, our pillared rGO membranes exhibit stable hexane permeance up to ∼2 L·m^–2^·h^–1^·bar^–1^ despite being ∼100–160 nm thicker (∼100–160 nm). Here, we have initially demonstrated how flux and rejection can be tuned by a combination of rGO membrane pillaring with PACs (of which a vast number are commercially available), followed by controlled reduction. At the same time, fabricating thinner membranes can produce large increases in flux. As shown in Figure S9, decreasing the membrane thickness from 130 to 70 nm increased the toluene permeance by approximately 3–4 times across all membrane types (rGO, rTBO-GO, and rSG-GO), while maintaining high rejection (95%) of polystyrene (2000 Da). These results are qualitatively consistent with the known microstructural changes occurring in rGO-based membranes upon thickness reduction.^6^

## Conclusions

This work demonstrates in detail that pillared rGO membranes are promising candidates for organic solvent separations in a range of both nonpolar and polar organic solvents. Pillared (rTBO-GO and rSG-GO) and nonpillared (rGO) membranes are able to perform organic solvent nanofiltration, with the performance being governed by the nature of the pillaring agent as well as the solvent viscosity and polarity. Intercalation followed by chemical reduction yielded stable membranes with approximately twice the permeance of nonpillared rGO in nonpolar solvents such as linear alkanes (*n*C_6_-*n*C_10_) and aromatics (C_6_–C_8_). All of the membranes showed excellent stability of fluxes and rejections in a range of organic solvents. The membranes exhibited tunable solute MWCOs of 500–600 Da in cross-flow measurements in a toluene solvent and permeances up to 1.5 L m^–2^ h^–1^ bar^–1^ (rSG-GO), with further increases in permeance upon thickness reduction of the membranes from ∼130 nm to ∼70 nm.

## Supplementary Material



## Notes

Detailed description of materials, membrane fabrication, pillaring and reduction procedures, characterization methods, nanofiltration testing protocols, cross-flow velocity, and pore size distribution calculations; Supplementary Tables (Tables S1–S2) summarize solute and solvent properties; Supplementary Figures (Figures S1–S9) depict the reduction mechanism, membrane fabrication procedures, characterization data, permeation data, and membrane permeation apparatus (PDF)
