# Supplementary material for: Pillared and Reduced Graphene Oxide Membranes for Organic Solvent Nanofiltration
Source: Ind Eng Chem Res. 2025 Sep 11;64(38):18817–25. doi: 10.1021/acs.iecr.5c01758 (PMC12464912; doi:10.1021/acs.iecr.5c01758)
Supplement: Supplementary file 1 [file ie5c01758_si_001.pdf]

Supporting Information

for

Pillared and Reduced Graphene Oxide Membranes for Organic Solvent Nanofiltration

Natechanok Yutthasaksunthorn[1], Kaung Su Khin Zaw[1,2], Scott A. Sinquefield[2], Sankar Nair[1,2*]

[1]*School of Chemical & Biomolecular Engineering, Georgia Institute of Technology, 311 Ferst Drive NW, Atlanta, GA 30332-0100, United States*

[2]*Renewable Bioproducts Institute, Georgia Institute of Technology, Atlanta, GA, 30332, USA*

* Corresponding author: sankar.nair@chbe.gatech.edu

Number of pages: 19

Number of supporting tables: 2

Number of supporting figures: 9

Experimental Methods

Materials and Chemicals

Sulfuric acid (98% H_2SO_4), graphite powder (synthetic grade, lateral size hydrochloric acid (HCl), sodium hydroxide (NaOH), potassium persulfate ($K_2S_2O_8$), phosphorus pentoxide (P_2O_5), potassium permanganate ($KMnO_4$), and pH 13 buffer were purchased from SigmaAldrich (Milwaukee, MI). A Thermo Scientific 7128 RO system produced deionized (DI) water. 30 nm pore size Polyvinylidene fluoride (PVDF) substrate and Polyether sulfone (PES) ultrafiltration membrane supports were obtained from Sterlitech Corp. Using DI water (conductivity = 4.5 µS, 25 °C) was supplied by the building. Pure ethyl alcohol, laboratory reagent hexane (≥ 95%), anhydrous heptane (99%) anhydrous octane (≥99%), reagent nonane (99%), anhydrous decane (≥99%), reagent benzene (≥99%), reagent grade toluene (99.5%), and reagent grade p-xylene (99%) for organic solvent nanofiltration measurement were purchased from SigmaAldrich (Milwaukee, MI). Powder Sodium Sulfate (Na_2SO_4, ≥99.0%, anhydrous), for salt rejection was purchased from SigmaAldrich (Milwaukee, MI) Hydriodic acid, 57 wt. % in H_2O for membrane reduction was purchased from SigmaAldrich (Milwaukee, MI)

GO Synthesis

GO was synthesized by a modified Hummers' method The detailed methods used for determining these characteristics are described in our prior work [1]. The prepared GO suspension was used for membrane fabrication.

Membrane fabrication

All membranes were prepared by a pressure-assisted filtration method onto PES/PVDF substrates (47-mm-diameter Millipore filters with 0.2-µm pore size), as shown in **Figure S1**. To obtain uniform and homogeneous membranes, the stock GO suspension was diluted to a concentration of

1 mg ml^{-1} before membrane preparation. To fabricate intercalated/pillared GO membranes, we used our prior method detailed in Wang *et al.*[2] for pillaring GO-TBO membranes and the method of Flores-Chaparro *et al.*[3] for pillaring GO-SG membranes, both illustrated in **Figure S1**. TBO- and SG-intercalated GO membranes were prepared by mixing the pillaring materials and GO suspension at a 1:1 weight ratio. The resulting membranes exhibited final weight percent loadings of 27 wt% for TBO-GO and 32 wt% for SG-GO, as calculated from Eq. S1. Since the vacuum filtration is carried out in a 'dead-end' mode, the degree of TBO incorporation in the membrane is determined from the difference in TBO concentration between the initial solution and the vacuum filtrate after membrane preparation (as measured by UV-vis spectrophotometry). After the vacuum filtration, the permeate was collected and its residual concentration was determined with a Cary-60 UV-vis spectrophotometer (Agilent Technologies). The TBO content in the membrane can then be determined by the following formula[2]:

$$\text{Pillaring Molecule Loading (wt.\%)} = \frac{c_B V_B - c_P V_P}{m_{GO} + m_{Pillaring}} \times 100\% \qquad \text{(Eq S1)}$$

Where c_B is the pillaring concentration in reagent B, V_B the volume of reagent B used in the preparation of the vacuum filtration solution, c_P the vacuum filtration permeate side TBO concentration measured by UV-vis, c_P the volume of the permeate, m_{GO} the mass of the GO deposited on the membrane surface (assumed identical to the mass of GO in the suspension) and $m_{Pillaring}$ the mass of the pillaring deposited on the membrane surface.

Reduction of the membranes was performed using hydriodic acid (HI) as reductant. HI is chosen for the tunable reduction process because: (1) it is a strong enough reducing agent that can operate at room temperature, and (2) compared to other commonly used reducing agents, such as hydrazine or sodium borohydride, it is generally considered to be a more cost-effective option and feasible to be scaled up. Initially, HI-vapor reduction processes at 80°C were utilized to reduce GO

membranes. However, the vapor reduction method exhibited difficulties in reproducibly controlling the membrane composition. Therefore, a modified HI reduction process was implemented (**Figures S2-S3**). A vacuum-assisted direct reduction method, conducted at room temperature, enabled successful control of the reduction degree and quality of the fabricated reduced graphene oxide (rGO) membranes with a range of hydrophobicity/hydrophilicity by reducing different fractions of hydrophilic group on the GO membrane layers. In this process, lower concentrations of the HI reductant (5.7 wt% aqueous), could be used. This method is also more scalable and amenable to use on a larger scale in the future.

Thinner Membrane Fabrication

Thinner GO and pillared GO membranes (~70 nm) were fabricated by vacuum-assisted filtration onto 47 mm PVDF membrane substrate. A GO suspension (1 mg/mL) was prepared as described previously and reduced using the modified HI reduction process. For the non-pillared membrane, 0.5 mL of the GO suspension (0.5 mg total) was diluted in 500 mL DI water and ultrasonicated prior to filtration. For pillared membranes (e.g., rTBO-GO), intercalants were added at a 1:1 weight ratio to GO (0.5 mg each) before dilution and sonication, following our previously reported method. After vacuum filtration, the deposited membranes were subjected to in-situ compaction (30 bar DI water) and then chemically reduced with 5.7 wt% aqueous HI at room temperature. This approach closely follows our previous thinning protocol in Zaw et al.[6] and was adapted to ensure consistency across reduced and pillared GO systems.

Determination of Molecular Weight Cut-Offs (MWCOs)

The Molecular Weight Cut-Off (MWCO) of each membrane was determined by estimating the molecular weight (MW) of a test dye molecule exhibiting a 90% rejection rate. For aqueous measurements, the experiments were conducted with a 0.3 mM dye concentration, at 25 °C and a

transmembrane pressure differential of 30 bar. The selected dyes (**Table S1**) for this analysis included Methyl Blue (800 Da, acidic), Congo Red (700 Da, basic), (600 Da, acidic), Allura Red AC (500 Da, acidic), and Cresol Red (400 Da, basic). To ensure accurate and consistent results, the membranes underwent cleaning procedures between measurements. Specifically, after exposure to basic dyes, the membranes were cleaned with a 1 N HCl solution, while exposure to acidic dyes was followed by cleaning with a 1 N NaOH solution. The cleaning process involved stirring for 30 minutes at room temperature for dead-end cell and running for at least 12 hours in the crossflow cell operation. UV-vis spectra for dye rejection calculation were collected with an Agilent 8453 UV-vis spectrophotometer (Agilent Technologies). For MWCO determination in toluene, we used a range of hydrocarbon-soluble molecules (**Table S1**) selected to span a broad range of molecular weights. The solutes included azobenzene (182.8 g mol^{-1}), Oil Blue N (378.5 g mol^{-1}), Oil Red O (408.5 g mol^{-1}), fullerene C_{60} (720.6 g mol^{-1}), fullerene C_{70} (840.8 g mol^{-1}), and polystyrene (2000 g mol^{-1}). Each solute was dissolved in toluene at a concentration of 20 ppm. These molecules enabled a systematic assessment of the membranes' molecular weight cut-off (MWCO) and rejection behavior in an organic solvent environment. The cleaning process involved washing and running with pure toluene for at least 12 hours in the crossflow cell operation.

Characterization

X-ray diffraction (XRD) measurements were performed using a Rigaku MiniFlex600 diffractometer equipped with Cu Kα radiation (λ = 1.5406 Å) in the 2θ range of 3° to 50°. The measurements were carried out with a step size of 0.01° and a scanning speed of 5°/min to ensure high-resolution peak detection. Due to the weak X-ray intensity observed for membranes fabricated from a 1 mg/L GO dispersion, membranes prepared with a 10 mg/L dispersion were used to amplify the signal for analysis.

To investigate the influence of different organic solvents (water, ethanol, hexane, toluene) both pillared and non-pillared GO membranes were tested. Prior to XRD analysis, the membranes were subjected to the following treatment:

1. Vacuum Drying: Membranes were kept under vacuum for over hours to remove interlayer water.
2. Solvent Immersion: Membranes were immersed in the selected solvents for over 1 day.
3. Drying Before Measurement: To avoid solvent diffraction effects, the membranes were removed from the solvents and air-dried for 2–5 minutes immediately before measurements.

This systematic procedure allowed for accurate characterization of the interlayer spacing (d-spacing) changes in both pillared and non-pillared membranes, providing insights into the structural response of the GO-based membranes under diverse solvent conditions. The average d-spacings were calculated according to Bragg's law. Elemental content, and carbon-oxygen containing groups in GO and intercalated GO membranes, before and after reduction, were compared using X-ray photoelectron spectroscopy (XPS). A Thermo K-Alpha XPS instrument (Al Kα radiation) with a flood gun was used to perform X-ray photoelectron spectroscopy (XPS). The C-C bond is considered to be hydrophobic, while C–O–C and O–C=O bonds are considered to be hydrophilic on a series of GO and intercalated GO membrane. Reduction of GO decreases the amount of carbon–oxygen groups (C–O–C, O–C= O). We track the "hydrophobic/hydrophilic ratio" by quantifying the ratio of areas of the C-C bond and the combined areas of the C–O–C and O–C=O bonds.

Permeation Measurements

To quantify the nanofiltration (NF) properties of the membranes, permeation measurements were conducted with pure water, a salt solution (0.01 M Na_2SO_4), aqueous dye solutions, hydrocarbon solute/ dye solutions, and a range of organic solvents (**Table S2**). Two sets of experiments are performed to study the reduction degree effect and the combination of intercalation and reduction effect at different transmembrane pressures (TMPs) at room temperature. The flux (J) at each TMP was calculated using Equation (2):

$$\text{Flux} \quad (J) = \frac{\Delta m}{A\,\rho\Delta t} \qquad \text{(Eq S2)}$$

whereΔ m is the mass of the permeate collected at each time (Δt), A is the effective area of the membrane, and ρ is the density of the permeate. For 0.01 M Na_2SO_4 rejection, the conductivities of feed and permeate were measured in order to obtain the salt rejection. Conductivity difference between feed and permeate is used to measure the concentrations of Na_2SO_4 mg/mL in the feed and permeate samples[1]. Dye rejection in Molecular Weight Cutoff characterization is measured by UV–vis spectrophotometry for the feed and permeate concentration. For all separation measurements of NF membrane, the rejection (R) can be calculated using Equation (3):

$$\text{Rejection } (R) = 1 - \left(\frac{C_p}{C_f}\right) \qquad \text{(Eq S3)}$$

where C_p and C_f are the permeate and feed sample concentrations.

Viscous Flow through the membranes as governed by the Hagen-Poiseuille Law the Hagen-Poiseuille law describes the relationship between permeate flux and several parameters as demonstrated by the following equation [4]:

$$J = \frac{\varepsilon\pi r_p}{8\mu\delta\tau}\Delta p \qquad \text{(Eq S4)}$$

Where J is the flux of water and organic solvents ($m^3m^{-2}s^{-1}$), Δp is the transmembrane pressure (Pa), ε is the surface porosity, r_p is the average pore radius (m), μ is the liquid viscosity (Pa·s), δ is the membrane thickness (m) and τ is the membrane tortuosity

The Hagen-Poiseuille equation can be modified into Eq. (S5) for 2D lamellar membranes

$$Flux = \frac{h^4 \Delta p}{12L^2 \eta \Delta x} \quad \text{(Eq S5)}$$

where h is the d-spacing between GO nanosheets, Δp is the transmembrane pressure, L is the modal lateral dimension of the GO nanosheets, η is the viscosity of the solvent and Δx is the thickness of the GO membrane.

Crossflow Velocity Calculation

$$Crossflow\ Velocity\ (cm/s) = \frac{Volumetric\ flowrate\ (cm^3/s)}{Sectional\ Area\ (cm^2)} \quad \text{(Eq S6)}$$

$$Sectional\ Area\ (cm^2) = Half\ Diameter\ (cm)\ \times Channel\ Height\ (cm) \quad \text{(Eq S7)}$$

Reynolds Number Calculation

$$Re = \frac{\rho u d_h}{\mu} \quad \text{(Eq S8)}$$

Where

ρ = Fluid density (kg/m^3)

u = Crossflow velocity (m/s)

d_h = Hydraulic diameter (m)

μ = Dynamic viscosity of the fluid (Pa·s)

$$d_h = \frac{2WH}{W+H} \quad \text{(Eq S9)}$$

W: Width of the channel (m)

H: Height of the channel (m)

The Reynolds number indicates whether the flow in the channel is laminar (Re<2000), transitional (2000<Re<4000), or turbulent (Re>4000). For membrane systems, laminar flow is typically observed due to low velocities and narrow channels. The Re value helps assess hydrodynamic conditions and mass transfer effects.

Example of crossflow velocity and Reynolds Number calculation

1. Crossflow cell operation for the measurements in this work ran with the flowrate of 190 mL/min which is equal to 3.17 cm^3/s

Height of crossflow cell channel (cm)	Half diameter of the effective area (cm)
0.19	2.35

The height and diameter of the crossflow cell are obtained from the manufacturer (Steritech) equipment drawing and details:

2. Sectional Area (cm^2) = 2.35 cm x 0.19 cm = 0.45 cm^2
3. $Crossflow\ Velocity\ (cm/s) = \frac{Volumetric\ flowrate\ (cm^3/s)}{Sectional\ Area\ (cm^2)} = \frac{3.17\ \text{cm3/s}}{0.45\ cm^2} = 7.06\ cm/s$
4. $d_h = \frac{2WH}{W+H} = \frac{2(2.35)(0.19)\ cm^2}{2.35+0.19\ cm} = 0.35\ cm$
5. $Re = \frac{\rho u d_h}{\mu}$ (example for hexane, the lowest viscosity solvent)

$$Re = \frac{\rho u d_h}{\mu} = \frac{\left(659\,\frac{\text{kg}}{\text{m}^3}\right)(0.0706\ m/s)(0.0035\ m)}{(0.00031\ Pas)} = 525$$

6. $Re = \frac{\rho u d_h}{\mu}$ (example for ethanol, the highest viscosity solvent)

$$Re = \frac{\rho u d_h}{\mu} = \frac{\left(789\,\frac{\text{kg}}{\text{m}^3}\right)(0.0706\ m/s)(0.0035\ m)}{(0.00104\ Pas)} = 187$$

The Reynolds numbers are hence in the range of 187-525 (laminar flow) based upon the solvents with the lowest (hexane) and highest (ethanol) viscosities as listed in **Table S2**.

Pore Size Distribution Calculation

Physical properties of toluene used for pore size distribution calculation:

Solvent	MW (g/mol)	dm (nm)	η (mPa s)	V_m $(cm^3 mol)^{-1}$	Density (g mL^{-1})
Toluene	92.12	0.70	0.55	106.8	0.866

Physical properties of solutes used for pore size distribution calculation:

Solute	MW (g/mol)	dm (nm)	Estimated Density (g mL^{-1})	V_m $(cm^3 mol)^{-1}$	Diffusivity in toluene (cm^2/s)
Azobenzene	182.23	0.788	1.18	154.4	1.90 x 10^{-5}
Oil Blue N	378.50	0.961	1.35	280	1.33 x 10^{-5}
Oil Red O	408.50	1.010	1.25	330	1.23 x 10^{-5}
Fullerene C-60	720.66	1.115	1.65	437	1.04 x 10^{-5}
Fullerene C-70	840.75	1.161	1.70	494	9.36 x 10^{-6}

The diameter of the solute was obtained using the following equations.

$$dm = 2\left(\frac{3V_m}{4\pi NA}\right)^{1/3} \quad \text{(Eq S10)}$$

Where V_m is the molar volume calculated from solvent density and NA is the Avogadro's number.

To correlate the MWCO data with pore size distribution, the rejections values of neutral solutes in toluene were used as input data into the pore flow model. [1]

The Hagen-Poiseuille equation for 2D membranes such as GO membranes describes the volumetric flux (Jv) through the membrane comprising uniform nanochannels as capillaries:

$$J_{vi} = \bar{v}.4r\frac{1-\sigma}{L} \quad \text{(Eq S11)}$$

Where the porosity $\sigma = \frac{L'}{L+L'}$ (assuming L' <<< L, σ is near zero), $\bar{v}$ as the average solvent flux in pores (m/s), r as the pore size radius (nm), and L as the length of the GO sheet (nm) (L ~ 200nm received from AFM data).

The local hindrance factors, $K_{i,d}$ and $K_{i,c}$, quantify the resistance of a specific solute or ion within a pore relative to free diffusion and convection in a bulk dilute solution. The hydrodynamic interaction of solute molecules with the interlayer walls and the steric restrictions of the interlayers resulted from these hinderances in the interlayer space. λ is the ratio of r_s (solute hydrodynamic radius) and r (effective pore radius). The hinderance factors are a function of λ. For transport in slit pores formed by 2D materials, Dechadilok and Deen provide the correlations of these factors for 0 < λ < 0.95.

$$H(\lambda) = 1 + \frac{9}{16}\lambda \ln \lambda - 1.19358\,\lambda + 0.4285\,\lambda^3 - 0.3192\,\lambda^4 + 0.08428\,\lambda^5 \qquad \text{(Eq S12)}$$

$$W(\lambda) = 1 - 3.02\,\lambda^2 + 5.776\,\lambda^3 - 12.3675\,\lambda^4 + 18.9775\,\lambda^5 - 15.2185\,\lambda^6 + 4.8525\,\lambda^7 \qquad \text{(Eq S13)}$$

The steric partitioning coefficient Φ(λ) = (1- λ) relates to the overall hindrance factors:

$$K_{i,d} = \frac{H(\lambda)}{\Phi(\lambda)} \qquad \text{(Eq S14)}$$

$$K_{i,c} = \frac{W(\lambda)}{\Phi(\lambda)} \qquad \text{(Eq S15)}$$

The real/intrinsic rejection of solute (s) by the membrane can be expressed analytically as derived by Nghiem et al.

$$R = 1 - \frac{\Phi\, K_c}{1-(1-\Phi)(\exp -Pe)} \qquad \text{(Eq S16)}$$

Pe is the dimensionless Peclet number for the solute :

$$Pe = \frac{K_c \bar{v}\, L}{K_s\, D_s} \qquad \text{(Eq S17)}$$

Where diffusivity, D of a solute with the radius r_s is calculated using the Wilke-Chang formula.[2]

$$D = 7.4 \times 10^{-4}\, T \frac{\sqrt{\varphi\, MW_{sol}}}{\mu\ V_m^{0.6}} \qquad \text{(Eq S18)}$$

Where MW_{sol} is the MW of solvent molecule, φ is a dimensionless solvent parameter, and V_m is the solute molar volume. Assuming R(r) is a continuous function of the pore radius, probability density factor, f (r) is used to describe the pore size distribution:

$$f(r) = \frac{1}{r\sqrt{2\pi b}} \exp - \frac{\left(\log\left(\frac{r}{r^*}\right)+\frac{b}{2}\right)^2}{2b} \qquad \text{(Eq S19)}$$

$$b = \log\left[1 + \frac{\sigma}{r^*}\right] \qquad \text{(Eq S20)}$$

To calculate the function f(r), the mean pore radius (r^*) and the standard deviation σ were calculated. The distribution function is truncated to rmax.

$$\frac{f'R(r)}{f\ R\ (r)} = \frac{1}{\int_0^{rmax} f(R) dr} \qquad \text{(Eq S21)}$$

The overall rejection over the pore radii 0 < r < rmax can be calculated using:

$$R = \frac{\int_0^{rmax} \frac{f' R\ (r) r^4\ R(r)}{\mu\ (r)\ dr}}{\int_0^{rmax} \frac{f' R\ (r) r^4}{\mu\ (r) dr}} \qquad \text{(Eq S22)}$$

Where the viscosity of the solvent is assumed constant.

Supplementary Tables

Table S1. Organic dye solutes used in this work.

Dye/ Solute	Solvent used in this work	MW (Da)	Solute Charge	UV-vis absorbance peak (nm)	Estimated Kinetic Diameter (Å)	Chemical structure
Cresol Red	Aqueous	404	Anionic	435	10–12	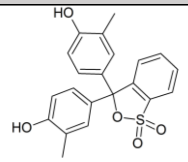
Allura Red	Aqueous	496	Anionic	501	11–13	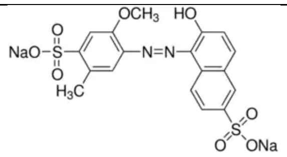
Congo Red	Aqueous	697	Anionic	492	13–15	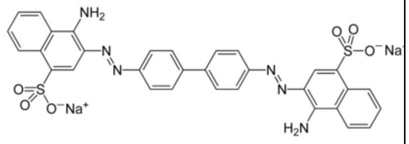
Methyl Blue	Aqueous	800	Anionic	583	14–16	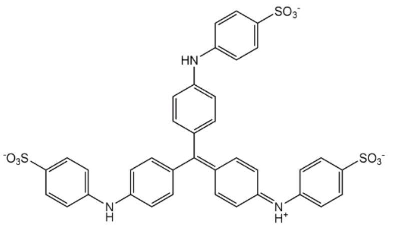
Azo-benzene	Toluene	182.83	Neutral	320	5–6	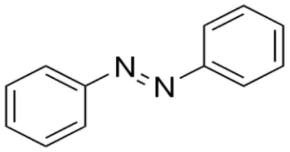
Oil Blue N	Toluene	378.51	Neutral	610	~10–12	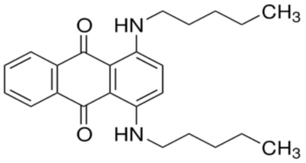

Oil Red O	Toluene	408.49	Neutral	518	~10–12	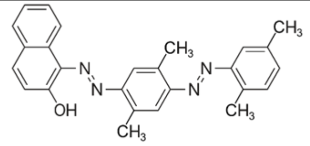
Fullerene C-60	Toluene	720.64	Neutral	330	~7.1 Å (exact known)	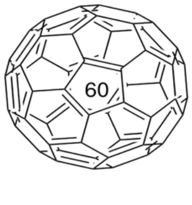
Fullerene C-70	Toluene	840.75	Neutral	379	~7.8 Å (elongated)	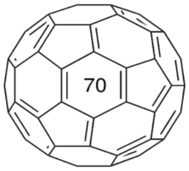
Poly-styrene standard	Toluene	2000	Neutral	NA	>20 Å (very flexible)	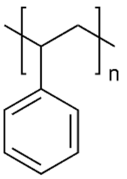

Table S2. Physical properties of organic solvents used in this work.

Solvent	Density @ 25 °C (g/mL)	Viscosity @ 25 °C (cP)	Relative Polarity	Kinetic Diameter (Å)
water	0.997	1.00	1.00	2.68
ethanol	0.789	1.04	0.65	4.3
n-hexane	0.659	0.31	0.09	4.3
n-heptane	0.6795	0.42	0.10	6.35
n-octane	0.703	0.51	0.10	4.3
n-nonane	0.718	0.67	0.01	4.5
n-decane	0.73	0.85	0.01	4.5
benzene	0.8765	0.603	0.11	5.8
toluene	0.87	0.56	0.10	5.8
p-xylene	0.861	0.64	0.07	5.8

Supplementary Figures

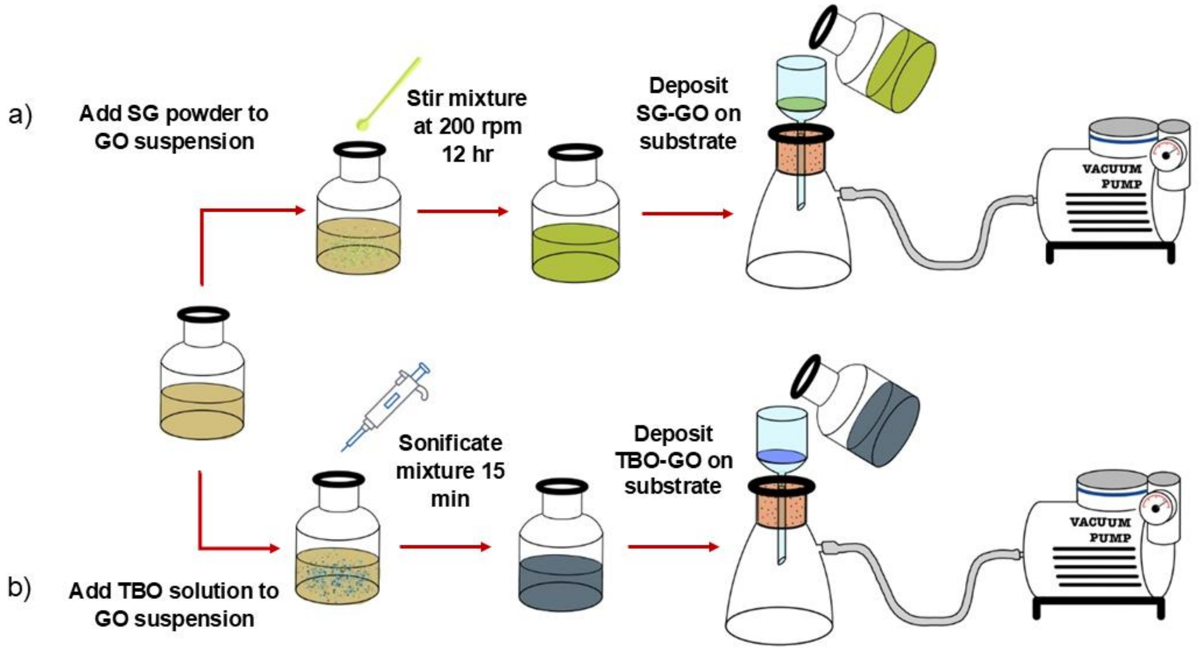

Figure S1. Fabrication of pillared membranes: (a) SG-pillared GO membrane and (b) TBO-pillared GO membrane prepared by vacuum filtration. (c) Corresponding weight percent loading of pillaring materials at 0.2:1, 0.4:1, 0.6:1, and 1:1 weight ratios (pillaring material:GO), calculated using Eq. S1.

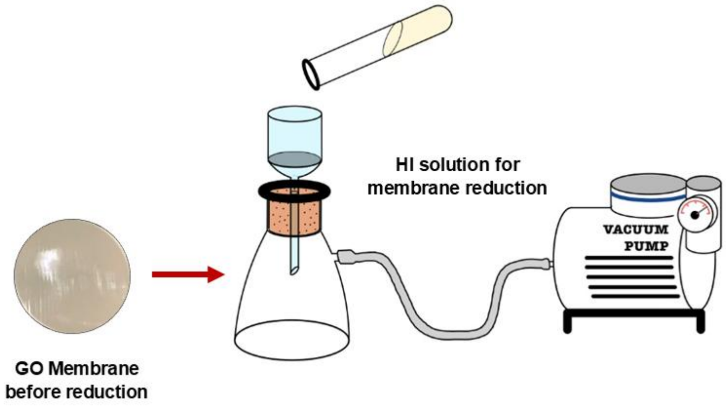

Figure S2. Modified vacuum-assisted HI reduction method for reduction degree control with HI concentration of 5.7 wt% (for this study).

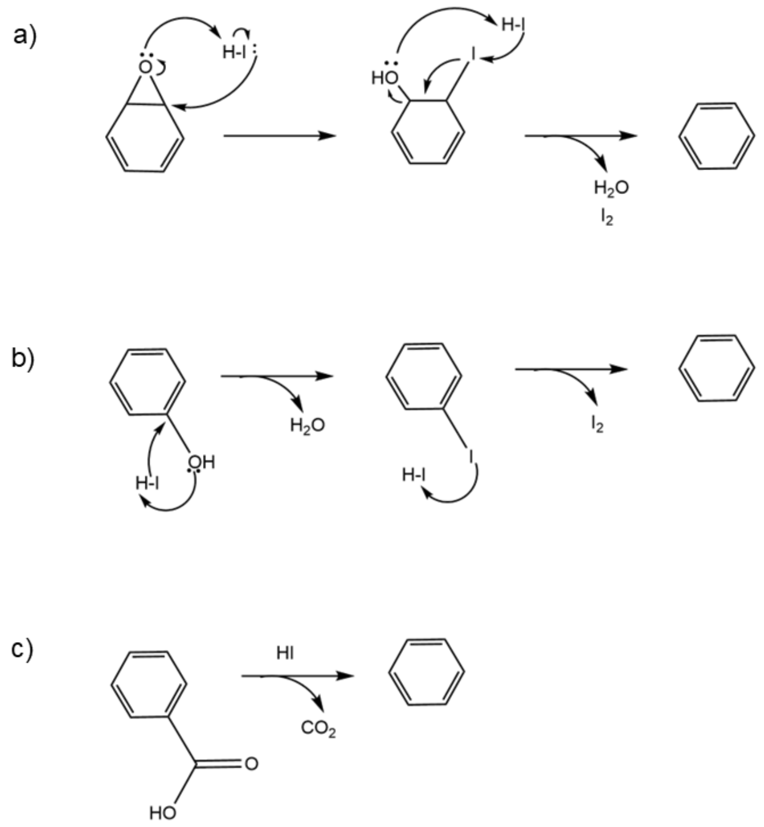

Figure S3. Mechanisms for the reduction of epoxide, hydroxyl, and carboxylic groups on GO surfaces with hydroiodic acid (HI) a) Route 1: Reduce epoxide group, b) Route 2: Reduce hydroxyl group, c) Route 3: Reduce carboxylic group.

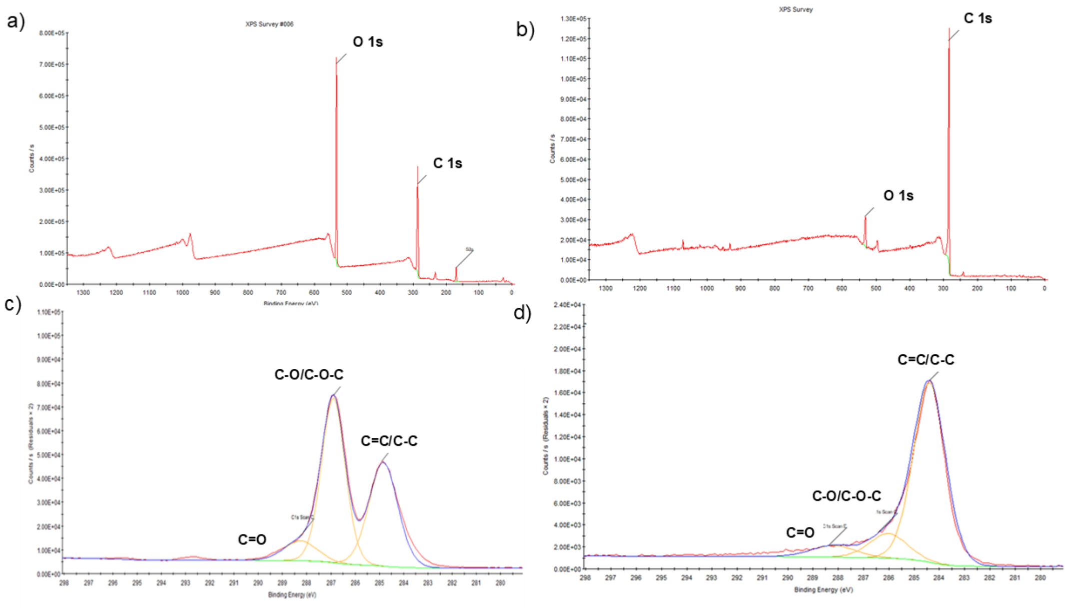

Figure S4. Example XPS spectra before and after reduction. (a) Survey spectrum of GO membrane. (b) Survey spectrum of rGO membrane. (c) C spectrum of GO membrane. (d) C spectrum of rGO membrane, showing detailed peak fitting.

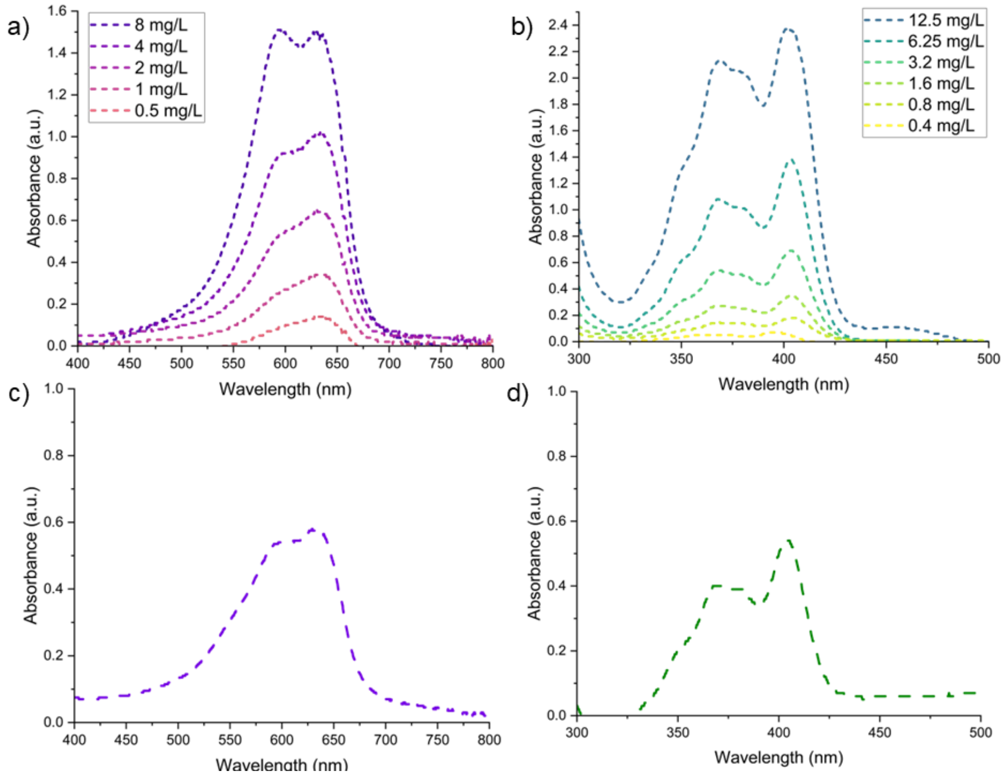

Figure S5. UV–vis absorbance spectra of (a) toluidine blue O (TBO) and (b) solvent green (SG) at varying concentrations in solution; and (c) TBO-GO and (d) SG-GO dispersions prior to membrane deposition.

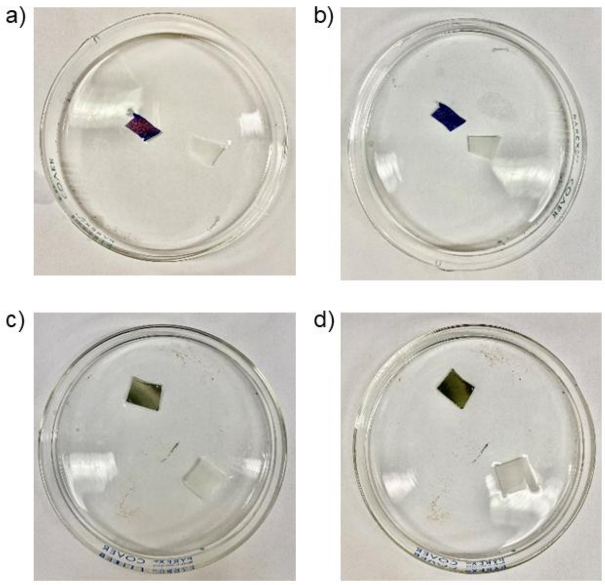

Figure S6. Example photographs of rTBO-GO and rSG-GO membranes before and after 30 days of immersion in water and ethanol. Black-colored samples are the rGO-based membranes, and clear-colored samples are the PVDF substrates as controls. (a) rTBO-GO at day 0, (b) rTBO-GO after 30 days, (c) rSG-GO at day 0, and (d) rSG-GO after 30 days. No visible delamination, swelling, or degradation was observed.

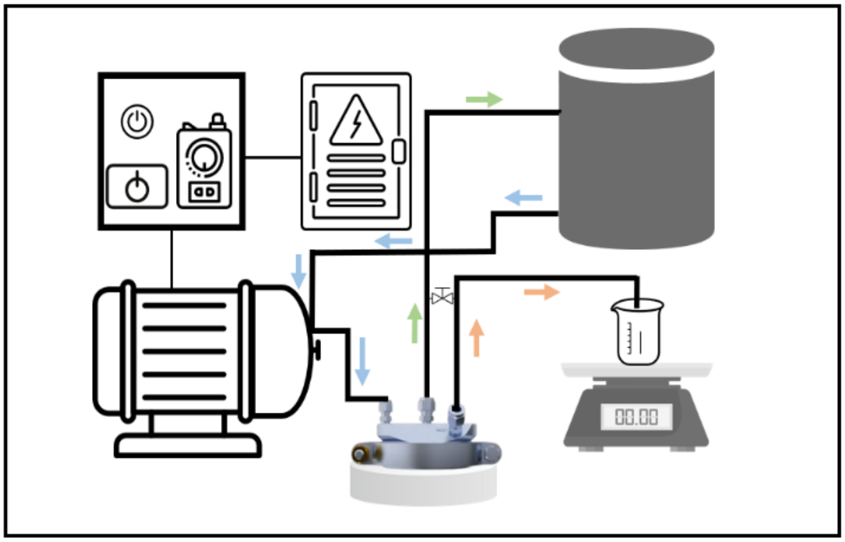

Figure S7. Schematic and photograph of the crossflow filtration setup used for membrane permeation measurements. The system consists of a power control box, variable frequency drive (VFD), and feed tank (top row), and a 3-phase motor connected to a 0.5 gal/min Catpump, CF047 membrane cell, and Ohaus 1200 g balance.

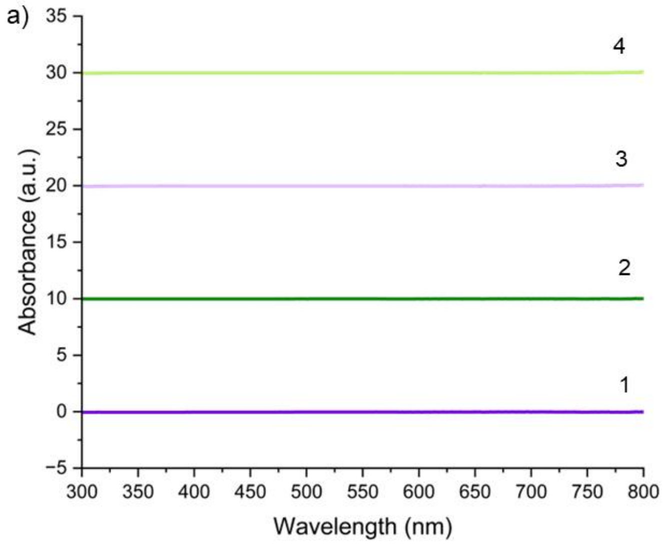

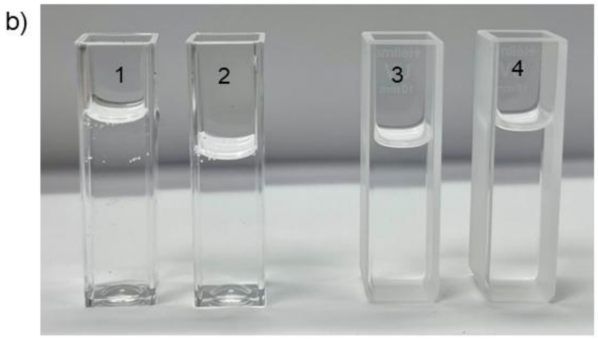

Figure S8. (a) UV–Vis spectra of cumulative permeates collected after continuous permeation of water or toluene through rTBO-GO and rSG-GO membranes in water and toluene (7 days each) at 30 bar. This includes the hydraulic conditioning of the as-made membranes for the first 2 days. The spectra are vertically stacked by 10 a.u. for clarity. (b) Photographs of the corresponding permeate samples used for UV-vis measurements (1–4), showing no visible color or pigmentation.

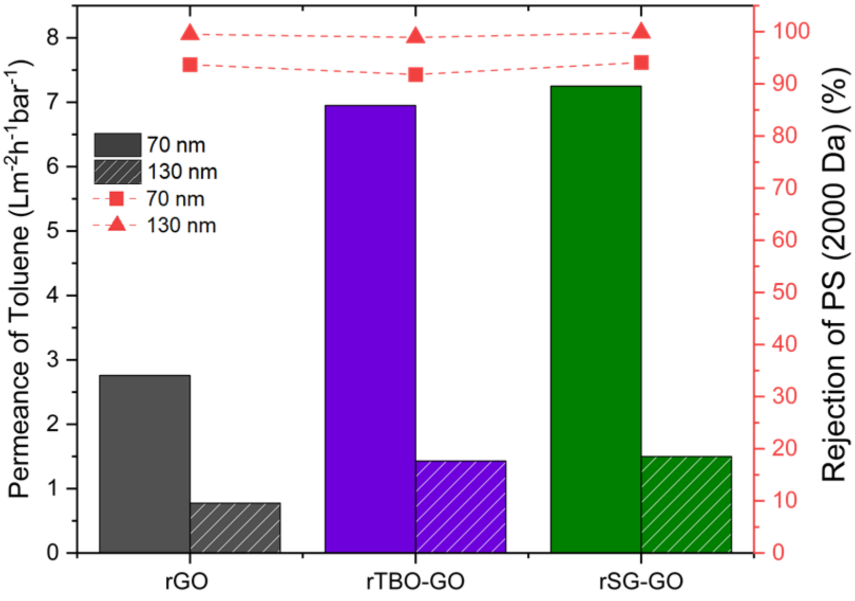

Figure S9. Effect of membrane thickness on toluene permeance (left axis) and PS (2000 Da) rejection (right axis).
